# A new technique to implant intracorneal ring-segments from the perilimbal region: one-year prospective pilot study report

**DOI:** 10.1186/s12886-024-03552-0

**Published:** 2024-07-16

**Authors:** Roberto Albertazzi, Carlos Rocha-de-Lossada, Roger Zaldivar

**Affiliations:** 1 Centro de Ojos Quilmes, Buenos Aires, 1865 Quilmes Argentina; 2 Instituto Zaldivar, Ciudad de Buenos Aires, 1865 Argentina; 3 Department of Ophthalmology, VITHAS Almería Hospital, Qvision, Almeria, 04120 Spain; 4 Department of Ophthalmology, Regional Universitary Hospital of Málaga, Granada, 18014 Spain; 5 Department of Surgery, Ophthalmology Area, University of Seville, Seville, 41012 Spain; 6 Department of Opthalmology, Vithas Malaga, Malaga, 29016 Spain

**Keywords:** Keratoconus, Intracorneal ring-segments, ICRS, Femtosecond laser

## Abstract

**Background:**

Recently a new surgical technique for intracorneal ring-segments (ICRS) assisted by femtosecond laser (FSL) called ByLimB was developed, involving the creation of the incision from a paralimbic region. This study aims to evaluate the safety and efficacy of the ByLimb technique following one year of follow-up.

**Methods:**

A prospective, single-center study was conducted at the Zaldivar Institute in Buenos Aires, Argentina. Keratoconus patients with indication for ICRS-FSL assisted procedure, operated with the ByLimb technique were included. By using the ByLimb technique, the ICRS are placed without affecting the tunnel’s roof, and the end of the ICRS is always away from the incision area. Visual acuity, topographic astigmatism, and the occurrence of complications were evaluated. Safety index was the main outcome and efficacy indes was a complimentary outcome.

**Results:**

A total of 17 eyes completed the 12-month follow-up period. The safety index was 1.10 (mean postoperative corrected distance visual acuity (CDVA) in decimal: 0.76/preoperative CDVA: 0.69), while the efficacy index was 0.89 (mean postoperative uncorrected distance visual acuity UDVA in decimal: 0.62/preoperative CDVA: 0.69). Mean preoperative astigmatism was 5.3 ± 2.3, decreasing twelve months after surgery at 2.1 ± 1.2 (*p* < 0.001). No eye loss lines of vision and no intraoperative complications were observed. During the first month after surgery, an improper positioning of the ICRS based on topographic assessment was detected in five cases. A second procedure was performed, which consisted of opening the incision and introducing a Sinsky hook, through which the ICRS was mobilized and placed in its correct position. No incisional alterations, signs of infection, anterior segment anomalies, or fundus alterations were observed.

**Conclusion:**

FSL-assisted ICRS implantation through the perilimbal region has demonstrated an adequate safety index one year post-surgery. Additionally, this technique has facilitated accurate realignment of ICRS during secondary surgical interventions within one-month post-surgery. While the current findings are promising, continued follow-up of these cases is warranted.

**Supplementary Information:**

The online version contains supplementary material available at 10.1186/s12886-024-03552-0.

## Background

Intracorneal ring segments (ICRS) are biocompatible synthetic elements implanted into the corneal stroma to modify its morphology [1]. Studies have demonstrated the efficacy and safety of ICRS in selected cases, particularly in addressing structural issues like primary ectasia (e.g., keratoconus or pellucid marginal degeneration) and secondary ectasia, as well as high and irregular postsurgical astigmatism [2, 3]. Alongside advancements in science and technology, including the emergence of novel therapeutic options like stem cell treatments [4], improvements in biomaterials, new ICRS designs, and refinements in surgical techniques, notably with the introduction of femtosecond laser (FSL) technology, have further enhanced the management of keratoconus [5, 6].

Despite significant progress, ICRS implantation, while generally safe, is not without its risks, including corneal infections, migration, or extrusion of the segments, primarily due to the creation of corneal incisions during the procedure, posing both short and long-term challenges [7, 8]. Motivated by these concerns, our group began to evaluate which aspects could be addressed to mitigate potential problems, whether by working on implant materials and design or on aspects related to surgical techniques. Several authors, such as Kozhaya et al. [9], Jacob et al. [10], highlight the advantages of allogenic intracorneal implants. Bteich et al. [11] for example, emphasize the potential of the corneal allogenic intrastromal ring segments (CAIRS) technique to create customized asymmetric implants that are prepared and tailored very specifically to each case. Likewise, this work highlights the role of the femtosecond laser in performing these tasks. Analyzing the possibilities that the femtosecond laser could offer, our team embarked on developing an experimental surgical approach for ICRS implantation aided by FSL technology, introducing the ByLimb technique in 2021 [12]. Notably, this technique involves creating the incision from a paralimbic region. This study aims to evaluate the safety and efficacy of the ByLimb technique following one year of follow-up.

## Methods

### Design and bioethics

A prospective, single-center, exploratory pilot study was conducted at the Zaldivar Institute in Buenos Aires, Argentina, focusing on keratoconus patients. The study, which was non-randomized and non-masked, adhered to the principles outlined in the Helsinki Declaration. Patients were fully informed about the study objectives and the risk/benefit comparison between the new surgical technique and the standard method, and they provided written consent. Approval for this research protocol was obtained from the Institutional Review Board of the Argentine Council of Ophthalmology.

### Population

Patients diagnosed with keratoconus grades 1, 2, or 3 according to the Amsler-Krumeich Classification [13], who had demonstrated stability for a minimum of 6 months without signs of topographic progression, and who were deemed suitable candidates for femtosecond laser (FSL)-assisted intracorneal ring segment (ICRS) implant surgery were eligible for inclusion. Patient enrollment commenced in July 2022 and concluded in December 2022. Contact lens wearers were required to discontinue use for at least 20 days before surgery. Patients with a history of glaucoma, retinal or strabismus surgeries, as well as those who had undergone pterygium surgery, conjunctival grafts, ocular surface or oculoplastic procedures, or any corneal refractive surgical procedures that could potentially impact the ICRS implantation, were excluded from the study.

### Parameters to be evaluated

Visual acuity was assessed using the Snellen scale and expressed as the logarithm of the minimum angle of resolution (LogMAR) for statistical analysis. Both uncorrected and corrected distance visual acuity (UDVA and CDVA respectively), corneal keratometry (K1: the flattest meridian; K2: the steepest meridian), and topographic astigmatism (measured using Tomey MS4; Nagoya, Japan) were evaluated. These parameters were compared with the baseline values (i.e., before pre-ICRS implantation) and those obtained at the 12-month follow-up.

Corneal and limbal health were assessed using slit-lamp examination with and without fluorescein both preoperatively and postoperatively during all follow-up visits. Wound healing at the implantation site was evaluated through slit-lamp examination and optical coherence tomography (OCT) imaging (Optovue Avanti; Optovue Inc., Fremont, CA, USA). Additionally, to screen for peripheral retinal degeneration, retinal tears, and retinal detachment, fundus examination was conducted using a binocular ophthalmoscope preoperatively and at 6 and 12 months post-surgery.

The primary outcome measure was the safety index, with the efficacy index serving as a complementary outcome measure. The safety index was calculated as the ratio of postoperative corrected distance visual acuity (CDVA) to preoperative CDVA, while the efficacy index was determined as the ratio of 12-month postoperative uncorrected distance visual acuity (UDVA) to preoperative CDVA. For calculation purposes, all visual acuity values were converted to decimal notation.

The occurrence of complications was meticulously assessed throughout the study. Any complication that arose was promptly documented, and the corresponding actions taken to address it were meticulously recorded. Complications were categorized based on their onset time into intraoperative, early postoperative, and late postoperative, as outlined below:

- Intrasurgical complications: These encompassed any deviation from the planned surgical technique, including alterations in the paralimbal incision area and the potential risk of corneal perforation.
- Early postoperative complications: These complications emerged within the first three months following surgery and were primarily associated with incision healing, overall corneal condition, and the accurate positioning of the ICRS along the intended axis.
- Late postoperative complications: These complications manifested after three months post-surgery and encompassed various issues such as alterations in the incision area, changes in the corneal tunnel (e.g., melting, migration, extrusion), and any other ocular abnormalities, including those affecting the fundus.

### Follow-up

The patients underwent monitoring in three distinct stages. The initial stage encompassed recruitment, selection, inclusion, and preoperative screening. This was succeeded by the surgical intervention stage. Subsequently, postoperative follow-up visits were scheduled at specific intervals, including day 1, 3, 7, 14, 30, 45, and 90 post-surgery. Additionally, routine follow-up examinations were conducted at 6 and 12 months post-surgery for each eye.

### Surgical technique

While a comprehensive description of the technique has been previously published, the key aspects are summarized below, supplemented by an accompanying video (Supplementary Video 1). Utilizing the LDV Z8 FSL (Ziemer, Bern, Switzerland), we initiated the procedure by creating a 360° corneal tunnel with an internal diameter of 5.4 mm and an external diameter of 7.0 mm. Notably, this tunnel included a wider region (0.2 mm inner and 0.2 mm outer) in the upper 60°, known as the landing zone. Subsequent to this, a corneal-limbal incision measuring 4.36 mm in length was precisely generated with the FSL, establishing connectivity with the bubbles formed in the landing zone. Throughout the surgical intervention, intraoperative OCT provided guidance. Upon connecting the two incisions using blunt-edged Mac Pherson forceps, the bubbles were liberated from the surgical plane. Following this step, programmed intracorneal ring segments (ICRSs), each 6 mm in diameter, were meticulously inserted into the corneal tunnel through the limbal incision with the assistance of Sinskey forceps. The surgical procedure concluded upon achieving proper placement of the ICRS. In this technique, it is necessary to perform two docking steps: the first docking is performed to create the corneal tunnel, while the second docking is conducted for the creation of the Limbal Incision.

### Characteristics of ICRS implanted

**Fig. 1:**
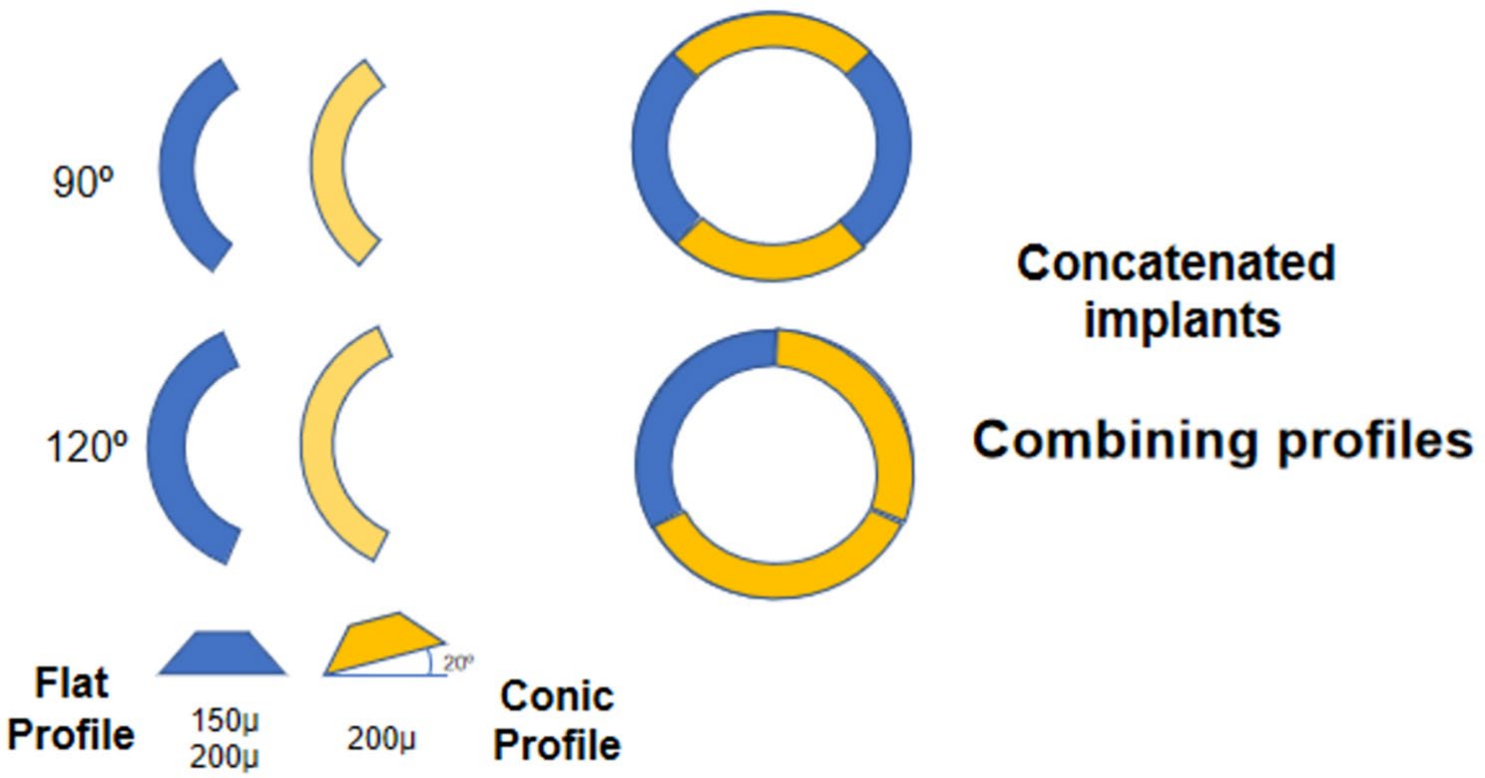
Concatenated intracorneal ring segments for combining profiles

This technique was developed to enable the implantation of ICRS with a 6 mm diameter from various commercial brands, featuring different terminations (straight and rounded) and angles (90°, 120°, and 150°). For this study, two types of ICRS were utilized, both manufactured by Keraring (Mediphacos-Belo Horizonte). One type was the Ferrara Ring, while the other consisted of concatenated ICRS models, combining various profiles. The main feature of this concatenated model is the presence of flat ends at 90° angles, which facilitates the insertion of one ICRS into the corneal tunnel by allowing the end of one segment to push the next, resulting in a concatenated implant formation. This approach combines ICRS with conical and flat profiles, as depicted in Fig. 1. To assess the safety of the technique, the results from both ICRS models were analyzed collectively.

### Statistics

To detect changes in the safety index with a confidence level of 95% and a statistical power of 80%, assuming a standard deviation of 0.5 and an effect size of 0.2, a sample size of 15 eyes was calculated. Considering a potential 10% loss to follow-up, a sample of 17 eyes was targeted. Descriptive statistical analysis and graphical representations were conducted using the XLMiner Analysis ToolPak software (Frontline Systems Inc., Incline Village, NV, USA). Data are presented as mean, standard deviation, and range. Normality of the study sample was confirmed using the Kolmogorov-Smirnov test. The paired t-test or one-way analysis of variance (ANOVA) was employed to compare means, with statistical significance set at *p* < 0.05. The study dataset is available as supplementary material.

## Results

**Fig. 2:**
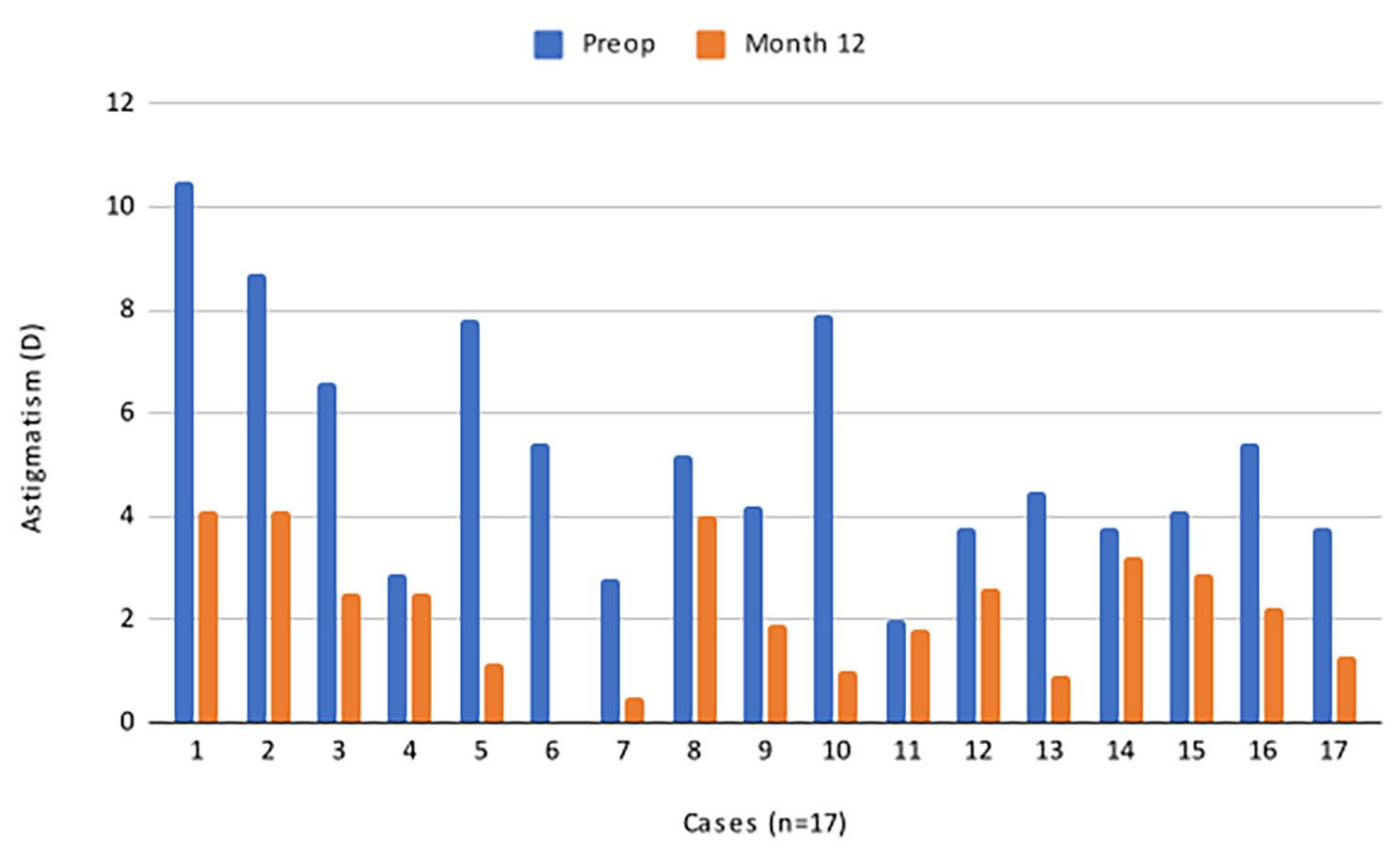
Astigmatism in eyes operated with the ByLimb technique, 12 months after surgery

A total of 17 eyes from 16 patients (4 women and 12 men) completed the 12-month follow-up period. The mean age was 27.1 ± 12.7 years (range: 14–55). Ten eyes were right (OD), and seven were left (OS). Visual performance and topographic astigmatism outcomes are presented in Table 1. It is noteworthy that there was a statistically significant reduction in astigmatism one-year post-surgery, with stability observed between months 3 and 12 postoperatively, as no statistically significant changes were noted. Figure 2 illustrates the astigmatism levels of each case before and one year after surgery.

**Table 1:** Refractive efficacy in eyes implanted with intracorneal ring segments (ICRS) using ByLimb technique. The arrows indicate which parameters were compared to determine changes between 3 and 12 months post-surgery; the result of the statistical test is shown in the last column

Parameters	Preop	1 months	3 months	6 months	12 months	*p*
**K1 (D)**	45.5 ± 4.3 (38.9–55.3)	45.9 ± 3.3 (39.4–54.1)	46.3 ± 3.2 (40.0–54.3)	46.6 ± 3.3 (39.8–54.4)	46.6 ± 3.4 (40.3–54.8)	**0.007**
**K2 (D)**	50.8 ± 4.2 (42.7–60.5)	49.1 ± 3.2 (42.7–56.8)	49.0 ± 3.3 (43.4–55.7)	48.7 ± 3.6 (41.3–56.5)	48.7 ± 3.6 (41.6–57.0)	**< 0.001**
**Astigmatism (D)**	5.3 ± 2.3 (2.0–10.5)	3.2 ± 1.0 (0.9–4.7)	2.7 ± 1.3 (0.5–5.8)	2.1 ± 1.0 (0.4–4.9)	2.1 ± 1.2 (0.0–2.2)	**< 0.001**
			→ → →			0.3
**UDVA (LogMAR)**	0.36 ± 0.4 (0.4–1.8)	0.34 ± 0.4 (0.4–1.8)	0.20 ± 0.2 (0.3-1.0)	0.22 ± 0.2 (0.2-1.0)	0.21 ± 0.2 (0.2-1.0)	**< 0.001**
			→ → →			0.7
**CDVA (LogMAR)**	0.16 ± 0.2 (0.1-1.0)	0.17 ± 0.2 (0.1–0.8)	0.15 ± 0.1 (0.1–0.8)	0.13 ± 0.1 (0.1–0.8)	0.12 ± 0.1 (0.1–0.7)	**0.21**
			→ → →			**0.8**

*UDVA: uncorrected distance visual acuity; CDVA: corrected distance visual acuity

**Fig. 3:**
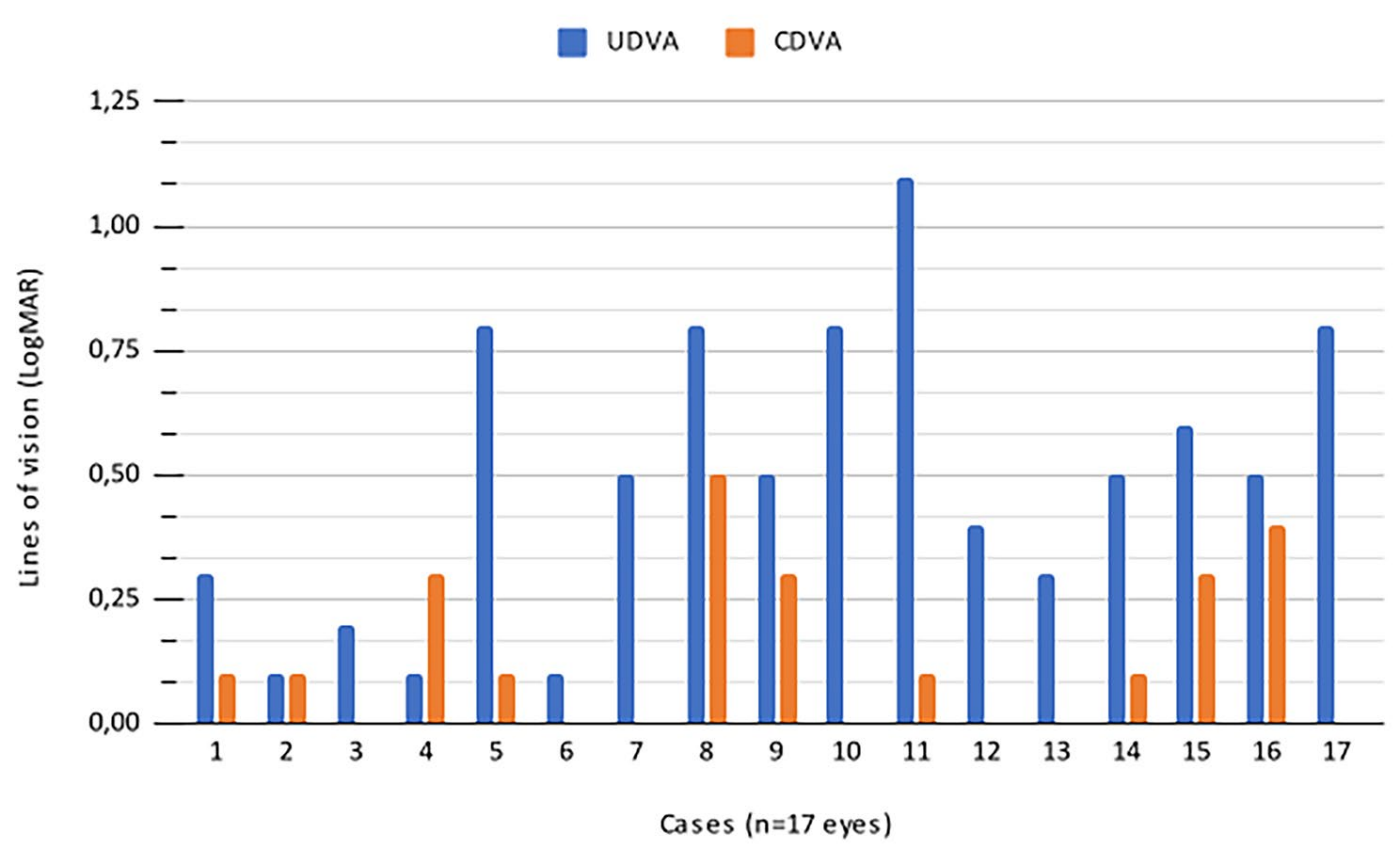
Changes in line of vision in keratoconus eyes operated with the Bylimb technique (comparison of UDVA, and CDVA before and 12 months after surgery). *UDVA: uncorrected distance visual acuity; CDVA: corrected distance visual acuity

Table 1 also demonstrates that UDVA exhibited statistically significant improvement one year after surgery, whereas CDVA remained stable, with no statistically significant changes noted. In terms of vision-related stability, both uncorrected and corrected distance visual acuities showed no statistically significant changes between month 3 and month 12 post-surgery. Notably, none of the cases experienced a loss of vision lines, as depicted in Fig. 3. Additionally, Fig. 3 illustrates that 7 eyes (41.1%), corresponding to cases 3, 6, 7, 10, 12, 13, and 17, maintained their CDVA lines unchanged 12 months after surgery. The remaining eyes demonstrated an increase in CDVA ranging from 0.1 to 0.5 on the LogMAR scale. The safety index was calculated as 1.10 (mean postoperative CDVA in decimal: 0.76/preoperative CDVA: 0.69), while the efficacy index was determined as 0.89 (mean postoperative UDVA in decimal: 0.62/preoperative CDVA: 0.69).

No intraoperative complications were observed. Early complications were detected in five cases (29.5%) during the first month after surgery, primarily due to improper positioning of the ICRS based on topographic assessment. To address this issue, a second procedure was conducted, involving remeasurement along the 0 to 180° axis and marking the desired position for the ICRS. The procedure, performed under topical anesthesia, consisted of reopening the incision (similar to a corneal flap) and using a Sinsky hook, maneuvering inside the corneal tunnel in order to mobilize and place the ICRS in its correct position. Among these cases, two involved Ferrara types ICRS, while three involved concatenated ICRS (these cases are further detailed in the supplementary video 1).

**Fig. 4:**
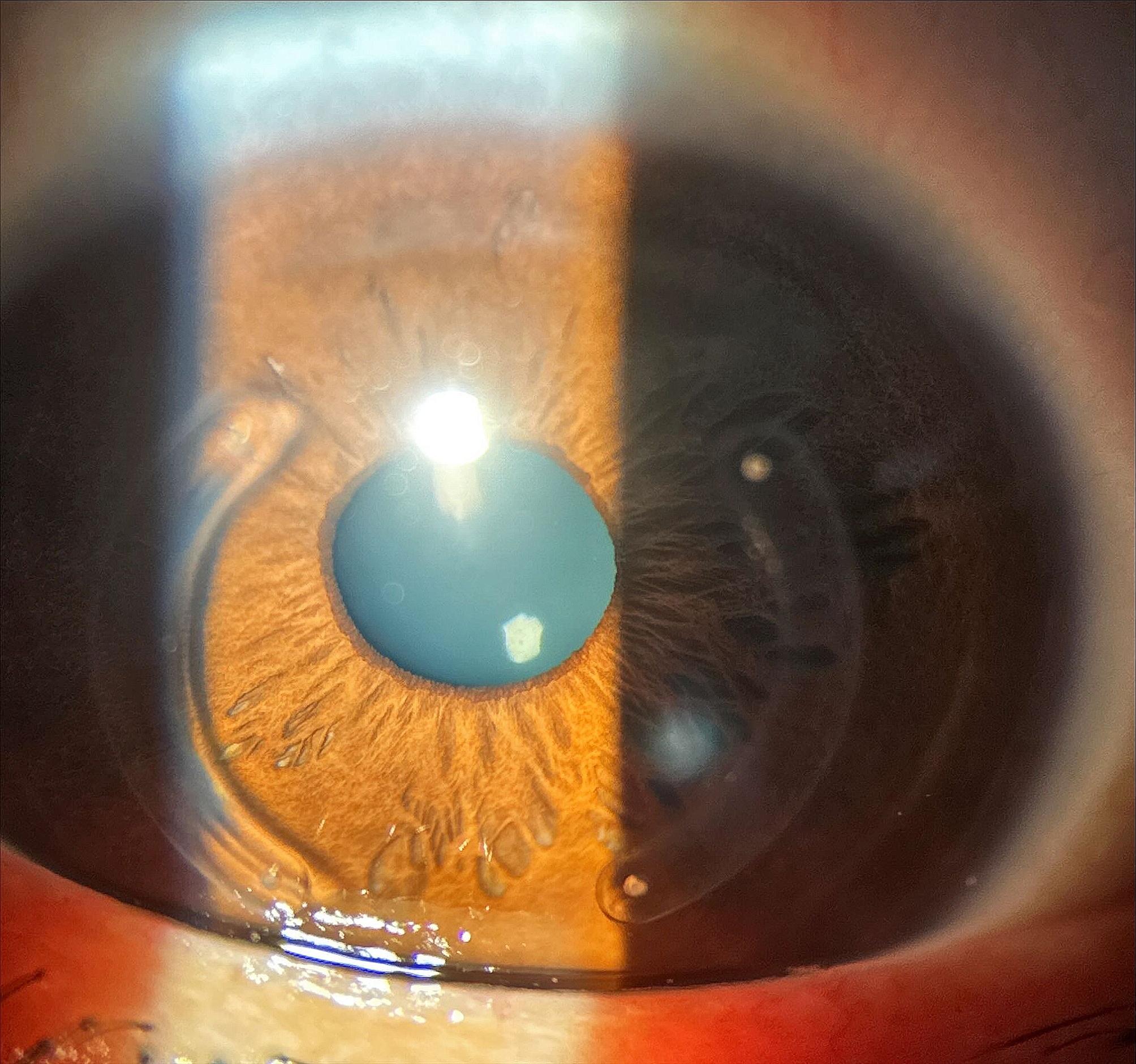
FIncision and healing area one year after surgery, after ICRS implantation using the ByLimb technique

**Fig. 5:**
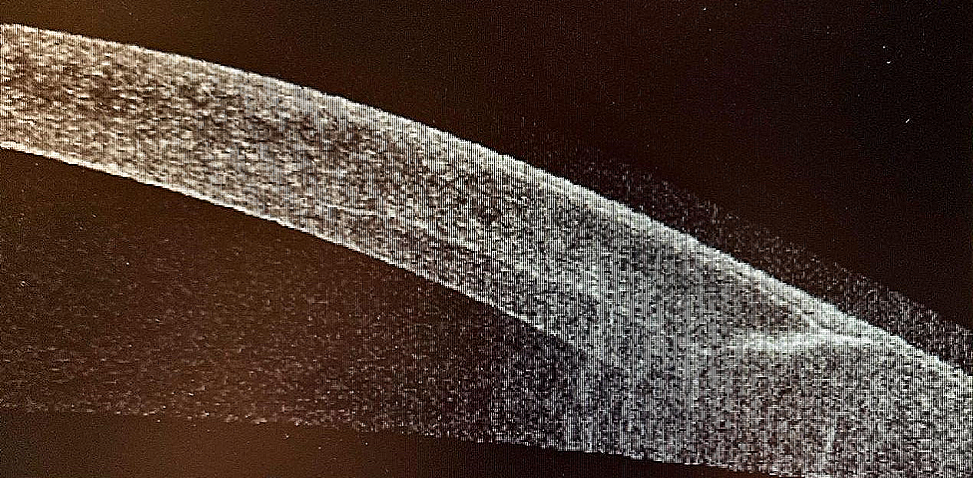
Same wound-healing zone observed in Fig. 4, but evaluated by OCT

**Fig. 6:**
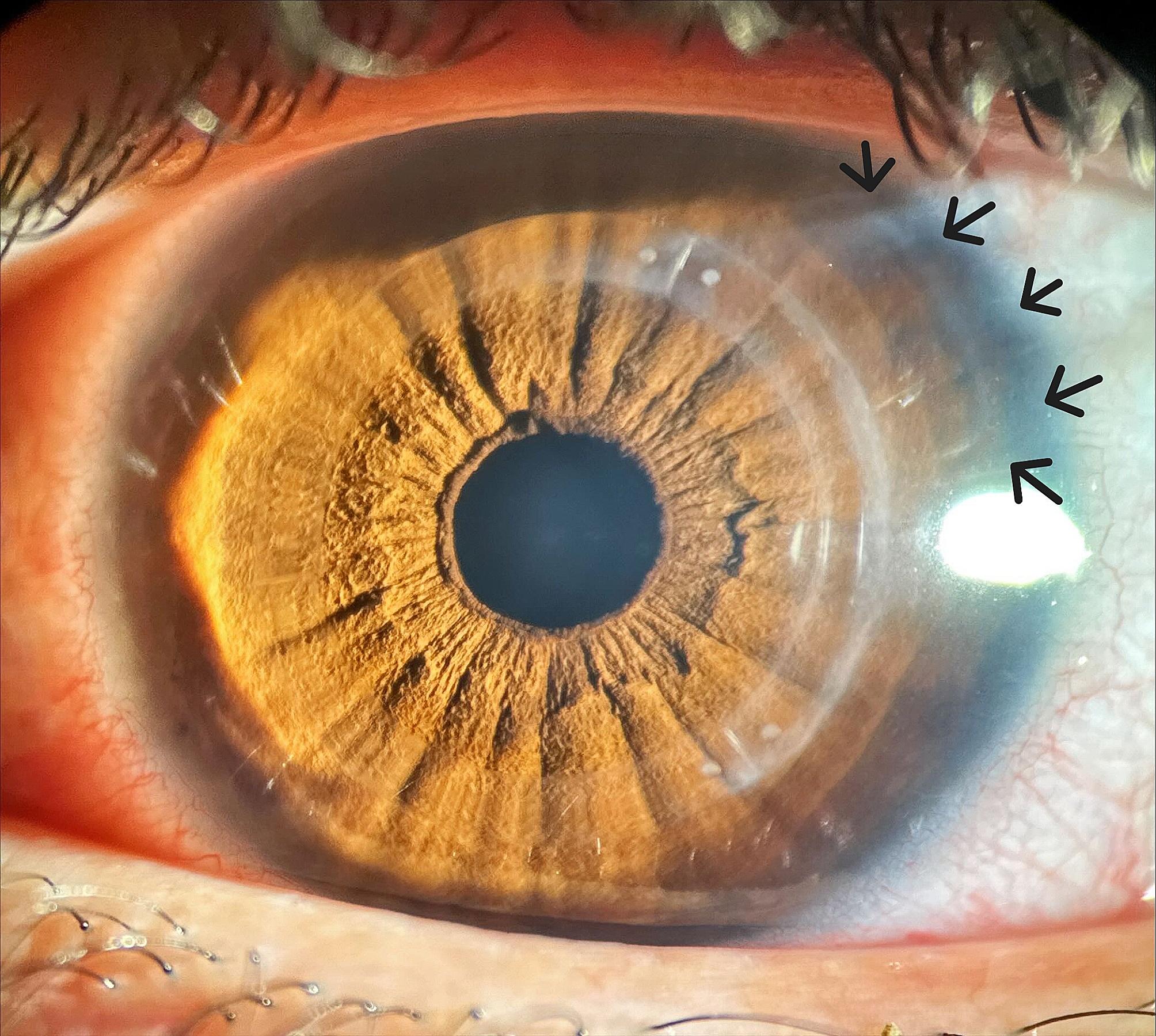
Aspect of the implantation site (arrows) one year after surgery, but in a case where three concatenated ICRS were implanted, using the ByLimb technique

**Fig. 7:**
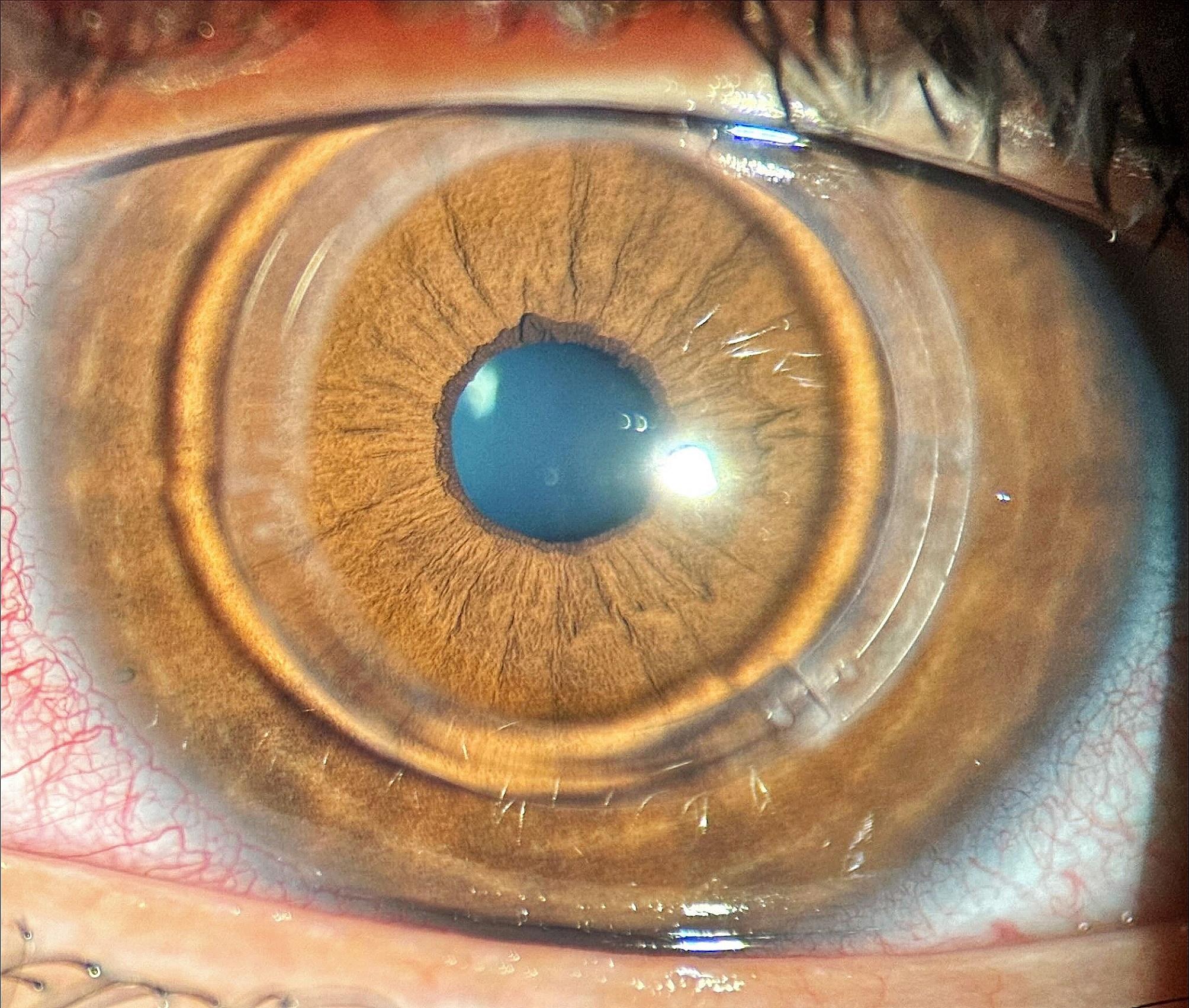
Same case as Fig. 6, but the picture shows the appearance of each of the three ICRS implanted in a concatenated fashion using the ByLimb technique

No late complications occurred as there were no observed incisional alterations, signs of infection, anterior segment abnormalities, or fundus alterations such as retinal tears or detachments. Additionally, there were no instances of displacement or extrusion of the implanted ICRS. Figure 4 illustrates the slight residual haze in the implantation zone one year post-surgery, while Fig. 5 depicts the same region evaluated using OCT. Figure 6 also shows the implantation site region used in the Bylimb technique, but in this case for three concatenated ICRS. In Fig. 7, the position of each of these three ICRS implants can be observed one-year post-surgery.

## Discussion

In this study, a novel FSL-assisted technique is introduced, enabling ICRS implantation through a limbal incision. Before this technique, all procedures described in the literature, whether manual or FSL-assisted, necessitated implantation through at least one corneal incision, with the segments’ ends typically positioned near the incision site.

One critical aspect of postoperative efficacy is ensuring the accurate positioning of ICRS [14]. The introduction of FSL techniques has undoubtedly facilitated and enhanced surgical outcomes. Nevertheless, it is widely acknowledged that during the docking process, alignment of astigmatism marking lines may sometimes be compromised, posing challenges to achieving precise alignment [15]. This discrepancy may result in the postoperative axis not aligning with the planned or pre-programmed axis. Furthermore, factors such as infection or corneal inflammatory processes can induce retrograde migration of ICRS within the stromal tunnel, leading to alterations in their position and refractive effects [7, 9, 16–18].

In our study, through meticulous follow-up, we observed the absence of inflammatory or infectious processes. Nevertheless, we noted an unexpected topographic outcome in nearly 30% of cases within the first month of follow-up, possibly attributed to alterations during the initial docking procedure. This complication provided practical validation of a theoretical advantage inherent in our technique: the ability to easily reposition the ICRS. By implanting the ICRS from a perilimbal region, akin to a corneal flap in refractive surgery, we could smoothly lift it during the postoperative period and reposition the ICRS as needed. This approach facilitates realignment of the ICRS during a secondary surgical intervention if required, thereby circumventing the need for re-entry through the corneal roof of the tunnel. Such re-entry could potentially heighten the risk of corneal damage or late complications [7, 8, 19, 20].

In this study, we assessed refractive efficacy parameters, including uncorrected distance visual acuity (UDVA), corrected distance visual acuity (CDVA), and astigmatism. We observed improvements in these parameters and their stability between months 3 and 12. However, as this study represents a pilot and exploratory investigation conducted for the first time in humans, our primary objective was to evaluate the safety of the technique. We are pleased to report satisfactory results, with no loss of vision lines in any eye, and nearly 60% of participants experiencing an improvement in CDVA. The safety index, a straightforward parameter comparing postoperative to preoperative CDVA, yielded a value of 1.1. Considering the significance of vision in decimal notation, achieving a safety index equal to or greater than 1 is deemed acceptable, further underscoring the positive outcome of our study.

Comparing our findings with existing literature proves challenging due to the absence of studies employing a similar technique. Nonetheless, for reference, Fernandez-Vega et al. [21] reported a safety index of 1.13 in a cohort of 409 patients with six months of follow-up, while Benlarbi et al. [22] more recently reported a value of 1.45, also with a six-month follow-up. It’s important to note that these studies differ from ours in various aspects, with the primary commonality being the evaluation of the safety index in keratoconus patients undergoing ICRS implantation. Despite these differences, achieving favorable safety outcomes encourages further investigation. We aim to explore the strengths and potential limitations of our technique in a larger sample size, employing different study designs, and soliciting the perspectives and experiences of other surgeons to assess the reproducibility of this novel approach to ICRS implantation.

We acknowledge the intricacies involved in implementing this technique, which necessitates advanced technology and surgical proficiency, particularly in the context of FSL ICRS implantation.Likewise, we understand that these technical aspects may currently be a limitation and an impediment to the widespread adoption of this technique, especially in countries with fewer economic resources and access to technology. Additionally, as this approach represents a novel advancement, surgeons may encounter a learning curve, albeit one that appears manageable. These considerations present partial limitations, exacerbated by the increased procedural time and costs compared to standard FSL techniques, as previously elucidated. Furthermore, it’s imperative to note the inherent constraint of a limited sample size in the present study, which is inherent to its status as an inaugural exploration of this innovative surgical modality.

Nevertheless, this technique offers notable advantages, foremost among them being the capability to create a 360° closed tunnel. This feature fosters innovation by facilitating exploration of novel ICRS designs, including concatenated combinations of profiles within a single tunnel, as well as injectable or articulated segments. Furthermore, by circumventing disruption to the roof of the corneal tunnel, the distal ends of the implants are positioned farther away from the incision site, thereby reducing the likelihood of wound healing complications—an issue we have previously documented [12]. This aspect holds particular significance, as common complications often stem from the insertion zone of the ICRS, where corneal epithelial ingrowth may precipitate localized corneal melting and segment migration, potentially culminating in implant extrusion in select cases [23, 24, 25]. The adoption of the present technique offers promise in mitigating and potentially preventing such wound-healing complications.

## Conclusions

FSL-assisted ICRS implantation, when performed through the perilimbal region, has demonstrated an adequate safety index one-year post-surgery. Nearly 60% of the eyes exhibited an improvement in visual acuity, both in UDVA and CDVA. Additionally, this technique has facilitated accurate realignment of ICRS during secondary surgical interventions within one-month post-surgery. While the current findings are promising, continued follow-up of these cases is warranted. Furthermore, future studies conducted across multiple centers and validated by independent surgeons are essential to corroborate the outcomes presented in this study.

### Electronic supplementary material

Below is the link to the electronic supplementary material.

Supplementary Material 1: Video 1. ByLimb surgical technique. This video summarizes the most relevant aspects of the present work, including an introductory rationale, a detailed description of the surgical procedure, and the presentation of results from several cases, involving both standard ICRS (Ferrara’s type) and concatenated implants.

Supplementary Material 2

## Data Availability

All data generated or analysed during this study are included in this published article [and its supplementary information files].

## Abbreviations

CDVA: Corrected distance visual acuity
FSL: Assisted by femtosecond laser
ICRS: Intracorneal ring-segments
OCT: Optical coherence tomography
UDVA: Uncorrected distance visual acuity
